# Hybrid Diagonal Approximation in Time‐Dependent Auxiliary Density Functional Theory

**DOI:** 10.1002/jcc.70210

**Published:** 2025-09-02

**Authors:** Kevin O. Pérez‐Becerra, Jesús N. Pedroza‐Montero, Mark R. Pederson, Luis I. Hernández‐Segura, Andreas M. Köster

**Affiliations:** ^1^ Departamento de Química Cinvestav Mexico; ^2^ Physics Department Central Michigan University Mt. Pleasant Michigan USA; ^3^ Department of Physics University of Texas at El Paso El Paso Texas USA

## Abstract

A hybrid diagonal approximation (HDA) for time‐dependent auxiliary density functional theory (TD‐ADFT) is presented. This newly implemented method allows the use of global and range‐separated hybrid functionals in TD‐ADFT for the calculation of vertical excitation energies and corresponding oscillator strengths. To preserve the exceptional computational efficiency and low‐order scaling of TD‐ADFT, only the diagonal elements of exact exchange are included in the TD‐ADFT matrices. For singlet excitations, this approximation reaches accuracies comparable to four‐center electron repulsion integral (ERI) implementations, albeit with a fraction of the computational cost. For triplet excitations, larger deviations are found with the HDA. Despite additional integral calculations, the low‐order scaling of TD‐ADFT is preserved with the HDA. We explain this by the intact index alignment between the ERIs and the excitation vectors, which remains unaltered in TD‐ADFT with the HDA.

## Graphical abstract

Our work demonstrates that time‐dependent auxiliary density functional theory (TD‐ADFT) in combination with the hybrid diagonal approximation (HDA) for global and range‐separated hybrid functionals is a promising approach for low‐order scaling electronic excitation energy calculations.

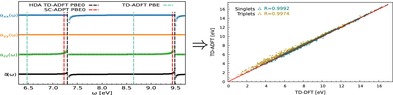

## 1 Introduction

Photoabsorption spectra are widely used in chemistry as an experimental technique to study the electronic structure of molecular systems. However, reliable assignments of spectral features can be challenging, especially in complex systems [1, 2]. In such cases, computational simulations play a crucial role in providing a better understanding of experimental observations. The development and implementation of efficient computational schemes for calculating molecular photoabsorption spectra is an active area of research [3, 4, 5, 6, 7, 8, 9]. One of the most popular methods is TD‐DFT [10, 11] which provides a good compromise between accuracy and computational efficiency. The RPA TD‐DFT as originally proposed by Casida [12, 13] is the most widely used TD‐DFT implementation in quantum chemistry. It involves the solution of an eigenvalue problem over a basis of single‐particle excitations. Other TD‐DFT formulations, such as those based on explicit time‐propagation techniques [2, 14], super‐operator formulation of TD‐DFT [15], and the Tamm‐Dancoff approximation (TDA) [16] have also been developed and successfully implemented. While accuracy remains a challenge, ongoing research is focused on improving the exchange‐correlation functionals and response kernels.

Nowadays, first‐principles electronic structure Kohn–Sham methods have reached an impressive level of computational efficiency, giving the possibility to calculate energies, structures and properties of molecules containing hundreds of atoms [17]. A key ingredient for this impressive performance of first‐principles calculations is variational density fitting [18] in combination with efficient electron repulsion integral (ERI) calculations [19, 20, 21]. A particular example for this development are response calculations in the framework of auxiliary density functional theory (ADFT) [22] employing auxiliary density perturbation theory (ADPT) [23]. ADPT combines the low memory demand of McWeeny's self‐consistent perturbation theory [24, 25, 26] with the favourable algebraic structure of an inhomogeneous equation system from the coupled‐perturbed Kohn‐Sham method [27], albeit with a much reduced dimension. Most recently, we have extended this approach to the RPA approximation of TD‐DFT. The resulting TD‐ADFT [28] shows excellent computational performance and scales sub‐quadratic with respect to the number of basis functions. Thus, TD‐ADFT is well suited for the calculation of excitation energies of large systems with many hundreds of atoms or for the calculation of photoabsorption spectra along Born‐Oppenheimer molecular dynamics trajectories. In comparison to TD‐DFT the TD‐ADFT excitation energies tend to be systematically larger for both RPA and TDA calculations, which slightly improves their agreement to experiment due to the systematic underestimation of excitation energies by the local density approximation (LDA) and generalized gradient approximation (GGA).

A severe limitation of the current TD‐ADFT implementation is its restriction to LDA and GGA functionals. It is well documented in the literature [29, 30, 31, 32, 33, 34, 35] that LDA and GGA can fail catastrophically for charge‐transfer (CT) excitations. Although, the development of exchange‐correlation functionals for the qualitative correct description of excitation energies is an ongoing active research field, [36, 37, 38, 39, 40, 41] it is generally admitted that Fock exchange is an essential ingredient for such new functionals. As a result, many works on the improvement of TD‐DFT excitation energies focus on global and range‐separated hybrid functional forms. Unfortunately, the inclusion of these hybrid functionals into ADFT or ADPT is not straightforward. Whereas efficient implementations for hybrid functionals into the self‐consistent field (SCF) ADFT energy calculations are now available [42, 43, 44] corresponding perturbation implementations in form of self‐consistent ADPT (SC‐ADPT) [45] are less satisfying in terms of computational performance. This is particularly true for the solution of the TD‐ADFT equation system. Therefore, it seems desirable to search for alternative approaches. An interesting proposal in this respect was recently given by Medves et al. [46]. Their hybrid diagonal approximation (HDA) incorporates only those ERIs from the Fock exchange response that do not alter the algebraic structure of the LDA or GGA TD‐DFT equation system. Therefore, the HDA is a promising approach to incorporate hybrid functionals, even if only approximately, into TD‐ADFT without jeopardizing its computational performance.

In their original implementation, Medves and co‐workers [46] have validated the HDA expression for global hybrid functionals, specifically for B3LYP [47, 48], showing a good quantitative agreement for optical properties with respect to experiment for medium‐sized systems. However, in global hybrid TD‐DFT calculations severe underestimations of Rydberg and charge‐transfer excitations have been observed [29, 37, 49]. Both deficiencies can be rectified for most systems by employing long‐range corrected functionals such as LC‐PBE, [36], LC‐BLYP [37], and CAM‐B3LYP [38]. The general consensus is that these functionals correct the asymptotic behavior of LDA and GGA functionals. Nevertheless, for core‐excitations short‐range corrected functionals are advocated, whereas inverse range‐separated functionals like HSE06 [50] are used to alleviate the self‐interaction errors present in GGA and global hybrid functionals [51]. To use these functionals in TD‐ADFT without jeopardizing its excellent computational performance we have developed a HDA variant of TD‐ADFT and extended it to range‐separated hybrid functionals, too.

The article is organized as follows. In the next section, we develop the working equations for HDA in the framework of TD‐ADFT and their extension to range‐separated hybrid functionals as implemented in the deMon2k code [52]. Special attention is given to the computationally efficient HDA specific ERI calculation. The computational methodologies alongside with the test set used in this article are described in Section 2. The results and discussion are presented in Section 3. They are divided into four subsections. In these subsections, we will discuss our validation tests for GGA, global hybrid and range‐separated hybrid functionals as well as for charge‐transfer excitations. Section 4 finishes with HDA benchmark calculations. Final conclusions are given in the last section.

## 2 Methodology

This work develops on the TD‐ADFT [28] implementation in deMon2k. In this method the same eigenvalue equation as in TD‐DFT needs to be solved:

(1) $$\Omega \text{F}=\omega^{2}\text{F}$$

The RPA Ω matrix and F eigenvectors for LDA and GGA functionals are defined as usual:

(2) $$\begin{array}{ll} \Omega_{ai,bj} & ={\left(A_{ai,bj}-B_{ai,bj}\right)}^{1/2}\left(A_{ai,bj}+B_{ai,bj}\right){\left(A_{ai,bj}-B_{ai,bj}\right)}^{1/2} \end{array}$$

(3) $$\begin{array}{ll} F_{ai} & =\sum_{b}^{uno}\sum_{j}^{occ}{\left(A_{ai,bj}-B_{ai,bj}\right)}^{-1/2}\left(X_{bj}+Y_{bj}\right) \end{array}$$

In Equation (3) $X_{bj}$ and $Y_{bj}$ are elements of the X and Y excitation vectors [12]. The difference to TD‐DFT arises from the definition of the super‐matrix elements $A_{ai,bj}$ and $B_{ai,jb}$. For LDA and GGA functionals, assuming a closed‐shell system, they are defined as [28, 53]:

(4) $$\begin{array}{ll} A_{ai,bj} & =\left(\varepsilon_{a}-\varepsilon_{i}\right)\delta_{ab}\delta_{ij}+B_{ai,bj}\; \end{array}$$

(5) $$\begin{array}{ll} B_{ai,bj} & =2\underset{\bar{k},\bar{l}}{\sum }\langle ai||\bar{k}\rangle O_{\bar{k}\;\bar{l}}\langle \bar{l}||bj\rangle \; \end{array}$$

In these equations, we use the conventional molecular orbital (MO) notation, namely *i, j* for occupied and *a, b* for unoccupied canonical MOs. The $\bar{k}$, $\bar{l}$ refer to primitive Hermite Gaussian auxiliary functions that are used in the self‐consistent field (SCF) procedure to expand the auxiliary density and ‖ is the Coulomb operator, $1/|{\vec{r}}_{1}-\vec{r_{2}}|$. The $O_{\bar{k}\;\bar{l}}$ matrix elements in Equation (5) are given by [28]:

(6) $$O_{\bar{k}\bar{l}}=G_{\bar{k}\bar{l}}^{-1}+\underset{\bar{m},\bar{n}}{\sum }G_{\bar{k}\bar{m}}^{-1}\langle \bar{m}|f_{xc}\left[\tilde{\rho }\right]|\bar{n}\rangle G_{\bar{n}\bar{l}}^{-1}$$

In Equation (6) $G_{\bar{k}\;\bar{l}}^{-1}$ refers to an element of the inverse Coulomb matrix [22]. The elements of the Coulomb matrix are given by: 
(7)

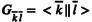

Furthermore, $f_{xc}\left[\tilde{\rho }\right]$ is the ADFT exchange‐correlation kernel [54, 55] defined as,

(8) $$f_{xc}\left[\tilde{\rho }\right]\equiv \;\frac{\delta^{2}E_{xc}[\tilde{\rho }]}{\delta \tilde{\rho }(\vec{r})\delta \tilde{\rho }(\vec{r}\prime )}$$

and $\tilde{\rho }(\vec{r})$ denotes the auxiliary density given by:

(9) $$\tilde{\rho }(\vec{r})=\underset{\bar{k}}{\sum }x_{\bar{k}}\;\bar{k}(\vec{r})$$

In Equation (9) $x_{\bar{k}}$ are the density fitting coefficients [18, 22]. The computational performance of TD‐ADFT is rooted in the index alignment of the super‐matrix elements defined in Equations (4) and (5) with the excitation vectors. As a result, ERI transformations from atomic to molecular orbitals can be completely avoided. Instead, the transformed excitation vectors are directly incorporated into the three‐center ERI calculation as shown in figs. 3 and 4 of reference [28]. When global hybrid functionals are used, the explicit definitions of the super‐matrix elements $A_{ai,bj}$ and $B_{ai,bj}$ change to:

(10) $$\begin{array}{c} \begin{array}{ll} A_{ai,bj} & =\left(\varepsilon_{a}-\varepsilon_{i}\right)\delta_{ij}\delta_{ab}+2\underset{\bar{k},\bar{l}}{\sum }\langle ai\|\bar{k}\rangle O_{\bar{k}\;\bar{l}}^{GH}\langle \bar{l}\|bj\rangle \\ & \;-c_{F}\underset{\bar{k},\bar{l}}{\sum }\langle ab\|\bar{k}\rangle G_{\bar{k}\;\bar{l}}^{-1}\langle \bar{l}\|ij\rangle \; \end{array} \end{array}$$

(11) $$\begin{array}{c} \begin{array}{ll} B_{ai,bj} & =\;2\underset{\bar{k},\bar{l}}{\sum }\langle ai\|\bar{k}\rangle O_{\bar{k}\bar{l}}^{GH}\langle \bar{l}\|bj\rangle -c_{F}\underset{\bar{k},\bar{l}}{\sum }\langle aj\|\bar{k}\rangle G_{\bar{k}\;\bar{l}}^{-1}\langle \bar{l}\|bi\rangle \; \end{array} \end{array}$$

In Equations (10) and (11), the $c_{F}$ coefficient is functional dependent as shown in Table S1 of the Supporting Information for functionals relevant to this work. The global hybrid (GH) $O_{\bar{k}\bar{l}}^{GH}$ matrix elements account for the exchange kernel adjustment according to the Fock exchange incorporation into the functional. These matrix elements are given by:

(12) $$\begin{array}{c} O_{\bar{k}\;\bar{l}}^{GH}=G_{\bar{k}\;\bar{l}}^{-1}+\underset{\bar{m},\bar{n}}{\sum }G_{\bar{k}\bar{m}}^{-1}\langle \bar{m}|f_{xc}\left[\tilde{\rho }\right]-c_{F}f_{x}\left[\tilde{\rho }\right]|\bar{n}\rangle G_{\bar{n}\bar{l}}^{-1} \end{array}$$

From Equation (2), it becomes obvious that building the Ω matrix with the above defined super‐matrix elements for global hybrids destroys the alignment of its elements with the excitation vector. As a consequence MO ERI transformations are required which significantly increase the computational demand. To overcome this computation bottleneck we propose here to apply the HDA [46] in the following form:

(13) $$\begin{array}{c} \begin{array}{ll} {\left(A-B\right)}_{ai,bj} & =\left(\varepsilon_{a}-\varepsilon_{i}\right)\delta_{ij}\delta_{ab}-c_{F}\underset{\bar{k},\bar{l}}{\sum }\langle ab\|\bar{k}\rangle G_{\bar{k}\;\bar{l}}^{-1}\langle \bar{l}\|ij\rangle \\ & \;+c_{F}\underset{\bar{k},\bar{l}}{\sum }\langle aj\|\bar{k}\rangle G_{\bar{k}\;\bar{l}}^{-1}\langle \bar{l}\|bi\rangle \\ & ≃\left(\varepsilon_{a}-\varepsilon_{i}-\Delta_{ai}^{GH}\right)\delta_{ij}\delta_{ab}\; \end{array} \end{array}$$

The new introduced HDA global hybrid super‐vector elements are defined as:

(14) $$\Delta_{ai}^{GH}=c_{F}\underset{\bar{k},\bar{l}}{\sum }\langle aa||\bar{k}\rangle G_{\bar{k}\;\bar{l}}^{-1}\langle \bar{l}||ii\rangle$$

Similar the (A+B) super‐matrix elements are approximated as:

(15) $$\begin{array}{c} \begin{array}{ll} & {\left(A+B\right)}_{ai,bj}=\left(\varepsilon_{a}-\varepsilon_{i}\right)\delta_{ij}\delta_{ab}+4\underset{\bar{k},\bar{l}}{\sum }\langle ai\|\bar{k}\rangle O_{\bar{k}\;\bar{l}}^{GH}\langle \bar{l}\|bj\rangle \\ & \;-c_{F}\underset{\bar{k},\bar{l}}{\sum }\langle ab\|\bar{k}\rangle G_{\bar{k}\;\bar{l}}^{-1}\langle \bar{l}\|ij\rangle -c_{F}\underset{\bar{k},\bar{l}}{\sum }\langle aj\|\bar{k}\rangle G_{\bar{k}\;\bar{l}}^{-1}\langle \bar{l}\|bi\rangle \\ & \;≃\left(\varepsilon_{a}-\varepsilon_{i}-\Delta_{ai}^{GH}\right)\delta_{ij}\delta_{ab}+4\underset{\bar{k},\bar{l}}{\sum }\langle ai\|\bar{k}\rangle O_{\bar{k}\;\bar{l}}^{GH}\langle \bar{l}\|bj\rangle \; \end{array} \end{array}$$

Thus, in our HDA variant of TD‐ADFT, the Ω matrix elements for a global hybrid functional are given by:

(16) $$\begin{array}{c} \begin{array}{ll} \Omega_{ai,bj} & \;={\left(\varepsilon_{a}-\varepsilon_{i}-\Delta_{ai}^{GH}\right)}^{2}\delta_{ij}\delta_{ab}\\ & \;+4{\left(\varepsilon_{a}-\varepsilon_{i}-\Delta_{ai}^{GH}\right)}^{1/2}\underset{\bar{k},\bar{l}}{\sum }\langle ai\|\bar{k}\rangle O_{\bar{k}\;\bar{l}}^{GH}\langle \bar{l}\|bj\rangle \\ & \;{\left(\varepsilon_{b}-\varepsilon_{j}-\Delta_{bj}^{GH}\right)}^{1/2}\; \end{array} \end{array}$$

In order to introduce range‐separated functionals into the TD‐ADFT equations it is necessary to partition the Coulomb operator into short‐range and long‐range contributions [56, 57]. A common ansatz is given by:

(17) $$\begin{array}{c} \frac{1}{\left|\vec{r}-\vec{r}\prime \right|}=\frac{1-erf(\omega |\vec{r}-\vec{r}\prime |)}{\left|\vec{r}-\vec{r}\prime \right|}+\frac{erf(\omega |\vec{r}-\vec{r}\prime |)}{\left|\vec{r}-\vec{r}\prime \right|}\; \end{array}$$

A graphical representation of this partition is given in Figure S1 of the Supporting Information. Another partition scheme commonly applied is the Coulomb attenuating method (CAM) [38] in which short‐range Fock exchange is considered, too. The CAM partition is defined as:

(18) $$\frac{1}{\left|\vec{r}-\vec{r}\prime \right|}=\frac{1-[\alpha +\beta \;erf(\omega |\vec{r}-\vec{r}\prime |)]}{\left|\vec{r}-\vec{r}\prime \right|}+\frac{\alpha +\beta \;erf(\omega |\vec{r}-\vec{r}\prime |)}{\left|\vec{r}-\vec{r}\prime \right|}$$

The CAM parameters are defined over the following intervals:

(19) 0≤α+β≤1,0≤α≤1,0≤β≤1

Based on these partitioning schemes we derived TD‐ADFT equations for range‐separated hybrid functionals employing the HDA. For the range‐separated (RS) HDA super‐vector elements we find analogous to Equation (14):

(20) $$\begin{array}{c} \Delta_{ai}^{RS}=c_{F}(\alpha \underset{\bar{k},\bar{l}}{\sum }\langle aa||\bar{k}\rangle G_{\bar{k}\;\bar{l}}^{-1}\langle \bar{l}||ii\rangle +\beta \underset{\bar{k},\bar{l}}{\sum }\langle aa\underline{\left|\right|}\bar{k}\rangle {\underline{G}}_{\bar{k}\;\bar{l}}^{-1}\langle \bar{l}\underline{\left|\right|}ii\rangle ) \end{array}$$

The here appearing α and β coefficients arise from the range separation schemes. We also included the global hybrid mixing coefficient $c_{F}$ such that Equation (20) can superseded Equation (14). Table S1 of the Supporting Information lists the functional dependent $c_{F}$, α and β coefficients. The short‐hand notation for the long‐range CAM operator is defined by:

(21) $$\underline{\left|\right|}=\frac{\alpha +\beta \;erf(\omega |\vec{r}-\vec{r}\prime |)}{\left|\vec{r}-\vec{r}\prime \right|}$$

For the efficient calculation of the long‐range three‐ and two‐center ERIs we refer the interested readers to reference [52]. The corresponding inverse Coulomb matrix elements are underlined. The RS Coulomb matrix elements are defined by: 
(22)

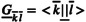

The TD‐ADFT super‐matrix elements for range‐separated hybrid functionals employing the HDA take the forms:

(23) $$\begin{array}{l} {\left(A-B\right)}_{ai,bj}=\left(\varepsilon_{a}-\varepsilon_{i}-\Delta_{ai}^{RS}\right)\delta_{ij}\delta_{ab}\; \end{array}$$

(24) $$\begin{array}{c} {\left(A+B\right)}_{ai,bj}=\left(\varepsilon_{a}-\varepsilon_{i}-\Delta_{ai}^{RS}\right)\delta_{ij}\delta_{ab}+4\underset{\bar{k},\bar{l}}{\sum }\langle ai\|\bar{k}\rangle O_{\bar{k}\;\bar{l}}^{RS}\langle \bar{l}\|bj\rangle \end{array}$$

Thus, for the corresponding Ω super‐matrix elements follows:

(25) $$\begin{array}{c} \Omega_{ai,bj}={\left(\varepsilon_{a}-\varepsilon_{i}-\Delta_{ai}^{RS}\right)}^{2}\delta_{ij}\delta_{ab}\\ \;+4{\left(\varepsilon_{a}-\varepsilon_{i}-\Delta_{ai}^{RS}\right)}^{1/2}\underset{\bar{k},\bar{l}}{\sum }\langle ai\|\bar{k}\rangle O_{\bar{k}\;\bar{l}}^{RS}\langle \bar{l}\|bj\rangle \\ \;{\left(\varepsilon_{b}-\varepsilon_{j}-\Delta_{bj}^{RS}\right)}^{1/2}\; \end{array}$$

The range‐separated $O_{\bar{k}\;\bar{l}}^{RS}$ matrix elements are given by:

(26) $$\begin{array}{c} O_{\bar{k}\;\bar{l}}^{RS}=G_{\bar{k}\;\bar{l}}^{-1}+\underset{\bar{m},\bar{n}}{\sum }G_{\bar{k}\bar{m}}^{-1}\langle \bar{m}|f_{x}^{SR}\left[\tilde{\rho }\right]+f_{c}\left[\tilde{\rho }\right]|\bar{n}\rangle G_{\bar{n}\bar{l}}^{-1} \end{array}$$

The here appearing short‐range (SR) exchange kernel is defined by:

(27) $$f_{x}^{\text{SR}}[\tilde{\rho }]\equiv \;\frac{\delta^{2}E_{x}^{\text{SR}}[\tilde{\rho }]}{\delta \tilde{\rho }(\vec{r}\;)\delta \tilde{\rho }(\vec{r}\prime )}$$

The short‐range exchange energy in Equation (27) has the form:

(28) $$\begin{array}{c} \begin{array}{ll} E_{x}^{\text{SR}} & \;=-\frac{3}{4}{\left(\frac{3}{\pi }\right)}^{\frac{1}{3}}\int {\tilde{\rho }}^{\frac{4}{3}}(\vec{r}\;)\;F_{x}(s)\\ & \;\left(1-\alpha -\frac{8}{3}a\beta \left[\sqrt{\pi }erf\left(\frac{1}{2a}\right)+2a(b-c)\right]\right)d\vec{r}\; \end{array} \end{array}$$

The a, b and c parameters are given by:

(29) $$a=\frac{\omega \sqrt{F_{x}}}{2\pi^{2/3}{\left(6\tilde{\rho }\right)}^{1/3}},\;b=\exp\left(-\frac{1}{4a^{2}}\right)-1,\;c=2a^{2}b+\frac{1}{2}$$

In these equations $F_{x}$ is the GGA enhancement factor and α and β are the previously introduced CAM coefficients.

### 2.1 HDA Correction Term Calculation

As Equations (16) and (25) show the difference between HDA and LDA or GGA TD‐ADFT matrix elements are the $\Delta_{ai}^{GH}$ or $\Delta_{ai}^{RS}$ super‐vector elements characteristic for HDA. Because their calculation involves three‐center ERIs a computationally efficient implementation is needed. For the sake of simplicity we restrict ourselves in the following to the $\Delta_{ai}^{GH}$ term. Extension to range‐separated hybrid functionals follow the same strategy as for the long‐range ERI calculations [45], that is, a modification of the basic integral calculation, and, therefore, is straightforward. To proceed, we expand the $\Delta_{ai}^{GH}$ super‐vector elements as:

(30) $$\begin{array}{ll} \Delta_{ai}^{GH}= & \;c_{F}\underset{\mu ,\nu }{\sum }c_{\mu a}c_{\nu a}\underset{\bar{k}}{\sum }\langle \mu \nu \|\bar{k}\rangle \underset{\bar{l}}{\sum }G_{\bar{k}\bar{l}}^{-1}\underset{\sigma ,\tau }{\sum }\langle \bar{l}\|\sigma \tau \rangle \;c_{\sigma i}c_{\tau i}\; \end{array}$$

**FIGURE 1:**
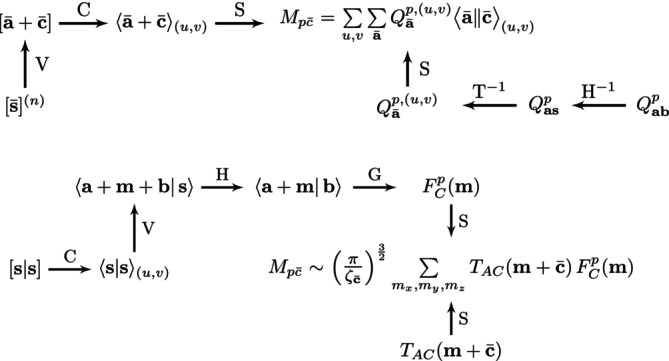
Pathway diagrams for near‐field (top) and far‐field (bottom) $M_{p\bar{c}}$ matrix elements ERI calculation.

In order to keep the HDA computational overhead to a minimum we avoid the explicit transformation of the three‐center ERIs. Instead, we calculate directly the $M_{p\bar{c}}=\langle p\;p\|\bar{c}\rangle$ matrix elements, with p being a canonical MO, according to the pathway diagrams in Figure 1. The elements of the rectangular matrix **M** with dimension $N_{bas}$ x $N_{aux}$, where $N_{bas}$ refers to the number of (contracted) orbital basis functions and $N_{aux}$ to the number of auxiliary basis functions, collect the final MO ERIs. To this end, the ERI calculation for the $M_{p\bar{c}}$ matrix elements is divided into near‐field and far‐field ERI calculations according to the double asymptotic ERI expansion [21]. The basic integrals for the near‐field ERI recurrence relation (see Figure 1 top) is calculated as:

(31) $${\left[\bar{\text{s}}\right]}^{\left(n\right)}=\frac{2\pi^{\frac{5}{2}}}{\sqrt{\zeta_{p}+\zeta_{\bar{c}}}}\frac{{\left(-2\alpha \right)}^{n}}{\zeta_{p}\;\zeta_{\bar{c}}}\;\kappa_{AB}\;F_{n}(T)$$

In Equation (31) $\zeta_{\bar{c}}$ is an exponent of a primitive Hermite Gaussian auxiliary function and $\zeta_{p}=\zeta_{a}+\zeta_{b}$ is the exponent of the primitive Gaussian basis set product function. The geometry factor $\kappa_{AB}$ is given by:

(32) $$\kappa_{AB}=e^{-\xi {\left(\vec{A}-\vec{B}\right)}^{2}}$$

with

(33) $$\xi =\frac{\zeta_{a}\;\zeta_{b}}{\zeta_{p}}$$

The argument of the Boys function,

(34) $$F_{n}(T)=\int_{0}^{1}t^{2n}e^{-Tt^{2}}dt\;\forall \;T\geq 0$$

is calculated as:

(35) $$T=\alpha \;{\left(\vec{P}-\vec{C}\right)}^{2}$$

Here $\vec{C}$ is the position vector for the center C at which the primitive Hermite Gaussian auxiliary function is located. The position vector $\vec{P}$ of the primitive basis function product and the exponent α are given by:

(36) $$\vec{P}=\frac{\zeta_{a}\vec{A}+\zeta_{b}\vec{B}}{\zeta_{p}}\;;\;\alpha =\frac{\zeta_{p}\;\zeta_{\bar{c}}}{\zeta_{p}+\zeta_{\bar{c}}}$$

The angular momentum index of the primitive basis integral given by Equation (31) in increased by the (primitive) vertical recurrence relation (V in the top pathway diagram of Figure 1):

(37) $${\left[\bar{\text{r}}+{\bar{\text{1}}}_{i}\right]}^{n}=(P_{i}-C_{i})\;{\left[\bar{\text{r}}\right]}^{n+1}+N_{i}(\bar{\text{r}}){\left[\bar{\text{r}}-{\bar{\text{1}}}_{i}\right]}^{n+1}$$

In Equation (37) $P_{i}$ and $C_{i}$ are components of the $\vec{P}$ and $\vec{C}$ position vectors and $N_{i}(\bar{\text{r}})$ takes the $i^{th}$ value of the angular momentum index $\bar{\text{r}}$. Once the vertical recurrence is finished the resulting primitive integral $\left[\bar{\text{r}}]=[\bar{\text{a}}+\bar{c}\right]$ is contracted. Because orbital exponents are included u,v‐scaled integrals are obtained:

(38) $$\begin{array}{c} {\left\langle \bar{\text{a}}+\bar{c}\right\rangle }_{\left(u,v\right)}=\overset{K_{a}}{\underset{k=1}{\sum }}\overset{K_{b}}{\underset{l=1}{\sum }}d_{ak}\;d_{bl}\frac{\zeta_{l}^{u}}{{\left(\zeta_{k}+\zeta_{l}\right)}^{v}}[\bar{\text{a}}+\bar{c}] \end{array}$$

In Equation (41) $d_{ak}$ and $d_{bl}$ are basis set contraction coefficients and $K_{a}$ and $K_{b}$ are the corresponding degrees of contraction, respectively. The resulting contracted ${\left\langle \bar{a}+\bar{c}\right\rangle }_{\left(u,v\right)}$ integrals are multiplied by the ${\left(-1\right)}^{\bar{c}}$ prefactor arising from the two‐center expansion of the Hermite Gaussian integral:

(39) $${\left\langle \bar{a}\|\bar{c}\right\rangle }_{\left(u,v\right)}={\left(-1\right)}^{\bar{c}}{\left\langle \bar{a}+\bar{c}\right\rangle }_{\left(u,v\right)}$$

The ${\left\langle \bar{\text{a}}\|\bar{c}\right\rangle }_{\left(u,v\right)}$ target integrals are directly used for the calculation of the $M_{p\bar{c}}$ matrix elements as the upper pathway diagram in Figure 1 shows. To do so, the $Q_{\mathrm{ab}}^{p}=c_{ap}\;c_{bp}$ matrix elements are transformed analogously to the density matrix elements in the original Coulomb vector integral implementation [20] employing the reverse horizontal ($H^{-1}$) and orbital transformation ($T^{-1}$) recurrence relations. Note, however, that these transformations have now to be performed $N_{bas}$ times, namely for all MOs p in the system.

The calculation of the far‐field ERI contribution to the $M_{p\bar{c}}$ matrix element is depicted in the pathway diagram at the bottom of Figure 1. This calculation starts with primitive overlap integral given by:

(40) $$[s|s]={\left(\frac{\pi }{\zeta_{p}}\right)}^{\frac{3}{2}}\kappa_{AB}$$

The (early) contraction [19] of these integrals yield:

(41) $${\left\langle s|s\right\rangle }_{\left(u,v\right)}=\overset{K_{a}}{\underset{k=1}{\sum }}\overset{K_{b}}{\underset{l=1}{\sum }}d_{ak}\;d_{bl}\;\frac{\zeta_{l}^{u}}{{\left(\zeta_{c}+\zeta_{l}\right)}^{v}}[\text{s}|\text{s}]$$

In the following vertical recurrence relation (V) from Obara and Saika [58] the angular momentum index in the bra is built up:

(42) $${\left\langle \text{a}+{\text{1}}_{i}|\text{s}\right\rangle }_{\left(u,v\right)}=(B_{i}-A_{i}){\left\langle \text{a}|\text{s}\right\rangle }_{\left(u+1,v+1\right)}+\frac{N_{i}(\text{a})}{2}{\left\langle \text{a}-{\text{1}}_{i}|\text{s}\right\rangle }_{\left(u,v+1\right)}$$

Note that the final angular momentum index in the bra contains not only the sum of the two basis function angular momentum indices a and b but also the asymptotic expansion order m that is set to 8 in our implementation [21]. The target modified overlap integral ⟨a+m|b⟩ is obtained from the horizontal recurrence relation (H) [59]:

(43) $$\left\langle \text{a}+\text{m}|\text{b}+{\text{1}}_{i}\rangle =\langle \text{a}+\text{m}|\text{b}\rangle +(A_{i}-B_{i})\langle \text{a}+\text{m}+{\text{1}}_{i}|\text{b}\right\rangle$$

At this point the gathering (G) step [21] is performed to build $F_{C}^{p}(m)$ according to:

(44) $$F_{C}^{p}(\text{m})=\frac{{\left(-1\right)}^{m}}{m_{x}!m_{y}!m_{z}!}\overset{\text{far}}{\underset{\text{a},\text{b}}{\sum }}Q_{\text{ab}}^{p}\langle \text{a}+\text{m}|\text{b}\rangle$$

In Equation (44) the sum over the atomic orbitals includes all pairs that are in the far‐field of center C. Furthermore, $\text{m}=m_{x}+m_{y}+m_{z}$ holds. Note that in both pathway diagrams of Figure 1 the integral recurrence relations are outside the MO loops. To complete the double asymptotic far‐field contribution to the $M_{p\bar{c}}$ matrix elements the diatomic Cartesian tensor $T_{AC}(m+\bar{c})$ as introduced by Stone [60] must be calculated:

(45) $$T_{AC}(m+\bar{c})\equiv {\left(\frac{\partial }{\partial C_{x}}\right)}^{m_{x}+{\bar{c}}_{x}}{\left(\frac{\partial }{\partial C_{y}}\right)}^{m_{y}+{\bar{c}}_{y}}{\left(\frac{\partial }{\partial C_{z}}\right)}^{m_{z}+{\bar{c}}_{z}}\frac{1}{\left|\vec{A}-\vec{C}\right|}$$

Finally, the double asymptotic contribution to the $M_{p\bar{c}}$ matrix element is calculated as:

(46) $$M_{p\bar{c}}∼{\left(\frac{\pi }{\zeta_{\bar{c}}}\right)}^{\frac{3}{2}}\underset{m_{x},m_{y},m_{z}}{\sum }T_{AC}(m+\bar{c})F_{C}^{p}(m)$$

Once all $M_{p\bar{c}}$ matrix elements are available the global hybrid HDA super‐vector elements are calculated as:

(47) $$\Delta_{ai}^{GH}=c_{F}\underset{\bar{k},\bar{l}}{\sum }M_{a\bar{k}}\;G_{\bar{k}\;\bar{l}}^{-1}M_{\bar{l}i}^{T}$$

Because the $\Delta_{ai}^{GH}$ calculation is outside the Davidson diagonalizer [53] random access memory (RAM) allocation is unproblematic.

### 2.2 Computational Details

The HDA scheme has been implemented in the developers version 6.3 of the deMon2k code [61]. For the global hybrid HDA validation the deMon2k PBE0 [62] and B3LYP implementations are used. For the corresponding validation of range‐separated hybrid functionals the deMon2k implementations of LC‐PBE, LC‐BLYP, CAM‐B3LYP and HSE06 are employed. As test inputs the subsets Q1, Q3 and Q5 from the quantum excited state (QUEST) [63, 64, 65] database are utilized. To this end, the first 30 singlet and triplet excitations from each of the 57 molecules of these QUEST database subsets are calculated employing the aug‐cc‐pVTZ [66] basis set and the GEN‐A2* [67, 68] auxiliary basis set. For triplet excitations, the nitroxyl molecule was excluded due to its triplet instability in most of the considered functionals. The TD‐DFT reference calculations were performed with the 5.0 version of Orca [69] without the HDA. To judge the benefit of global and range‐separated hybrids for CT excitation we performed PBE0, B3LYP, LC‐PBE, LC‐BLYP and CAM‐B3LYP HDA TD‐ADFT calculations employing the Q6 and Q7 subset of the QUEST database. The obtained results are compared with corresponding CC3 calculations from the literature [70, 71]. Finally, to assess the computational performance of HDA TD‐ADFT, serial benchmark calculations of linear alkane chains with the PBE0/6‐31G*/GEN‐A2* level of theory were carried out, too.

## 3 Results and Discussion

### 3.1 GGA Functionals

**FIGURE 2:**
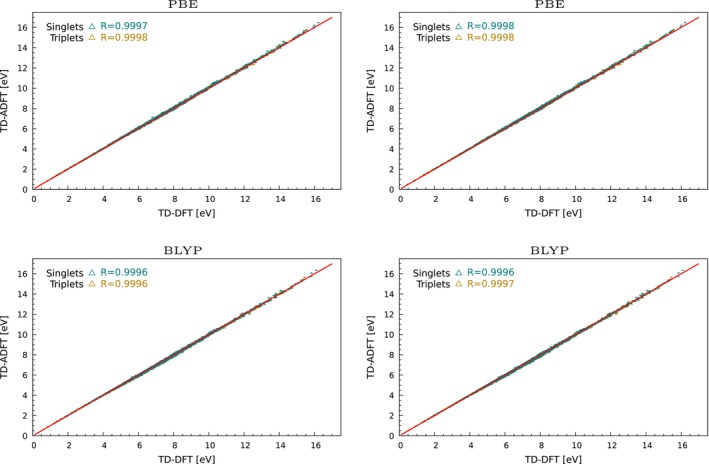
Linear correlation between TD‐DFT and TD‐ADFT excitation energies for RPA (left) and TDA (right) GGA calculations.

TD‐DFT implementations with LDA and GGA functionals have a long history in the deMon code development [53, 72, 73, 74]. Most recently, the TD‐ADFT deMon2k implementation was validated by Hernández‐Segura et al. [28] for LDA and GGA functionals. Good agreement with deviations of around 0.1 eV between TD‐DFT and TD‐ADFT results was found for both singlet and triplet excitations. For completeness we present a GGA validation again, now however with the Q1, Q3, and Q5 subsets from QUEST database. Figure 2 shows the corresponding correlation plots between TD‐DFT and TD‐ADFT for PBE [75] and BLYP [76, 77, 78] RPA and TDA excitation energies. As this figure shows excellent correlation for RPA and TDA excitations are obtained. The corresponding mean average error (MAE), mean signed errors (MSE) and root mean square error (RMSE) are depicted in Figure 6. For PBE MAEs and MSEs below 0.05 eV are found. For the BLYP functional slightly larger MAEs and RMSEs are obtained. Note that the MSEs for BLYP are positive (Figure 6), different to all other functionals. We assign this to the different B88 implementation in deMon2k that incorporates a cutoff in order to avoid violation of the Lieb‐Oxford bound. The standard deviation (STDE) of errors in Figure 6 combined with the histograms in Figure S2 of the Supporting Information show the error spread for the PBE and BLYP calculations. For PBE a clustering between −0.1 eV and 0.1 eV is found, whereas for BLYP the clustering is between −0.1 eV and 0.2 eV. The STDEs are in the range of 0.02 eV for both functionals and are similar for singlet and triplet excitations.

### 3.2 Global Hybrid Functionals

Hernández‐Segura et al. [28] showed that the TD‐ADFT excitation energies coincide with the poles of the average dynamic polarizability:

(48) $$\bar{\alpha }(\omega )=\frac{1}{3}[\alpha_{xx}(\omega )+\alpha_{yy}(\omega )+\alpha_{zz}(\omega )]$$

**FIGURE 3:**
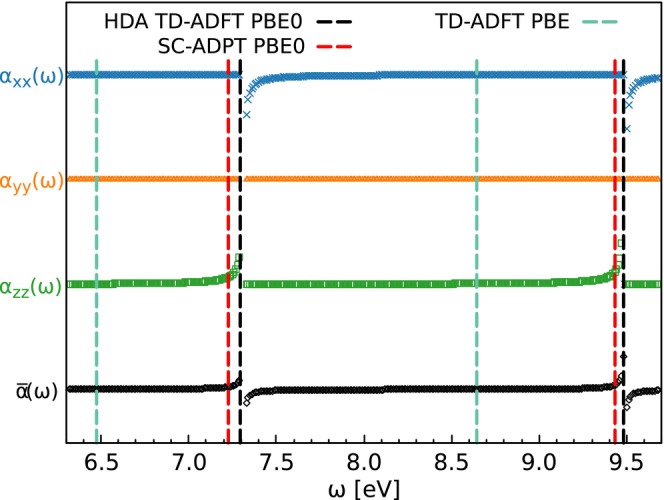
HDA PBE0 dynamical polarizabilities of ${\text{H}}_{2}\text{O}$ calculated with aug‐cc‐pVTZ and GEN‐A2* auxiliary basis set. The corresponding HDA TD‐ADFT excitation energies are given by the black vertical dashed lines. For comparison the SC‐ADPT poles (red vertical dashed lines) and TD‐DFT PBE excitation energies (green vertical dashed lines) are given, too.

Therefore, it seems pertinent to investigate how this relation is affected when the HDA is applied. To this end, we implemented the dynamic polarizability calculation with the HDA in deMon2k. Figure 3 shows the HDA PBE0 dynamic polarizabilities for the ${\text{H}}_{2}\text{O}$ molecule employing the aug‐cc‐pVTZ basis in conjunction with the GEN‐A2* auxiliary basis set. The corresponding HDA TD‐ADFT PBE0 excitation energies are given by the black dashed vertical lines. As Figure 3 shows the HDA TD‐ADFT excitation energies coincide perfectly with the corresponding average HDA dynamic polarizability poles that are given by the black curves in the bottom of the Figure 3 labeled $\bar{\alpha }(\omega )$. For comparison we also included in Figure 3 the exact PBE0 SC‐ADPT poles as red dashed vertical lines and TD‐ADFT PBE excitation energies as green dashed vertical lines. Comparison of the latter ones with the HDA TD‐ADFT PBE0 excitation energies illustrate the large impact that the Fock exchange in global hybrid functionals has on the resulting excitation energies. On the other hand, the difference between the SC‐ADPT and HDA PBE0 poles of the dynamic polarizability and, thus, of the corresponding excitation energies, is rather small.

**FIGURE 4:**
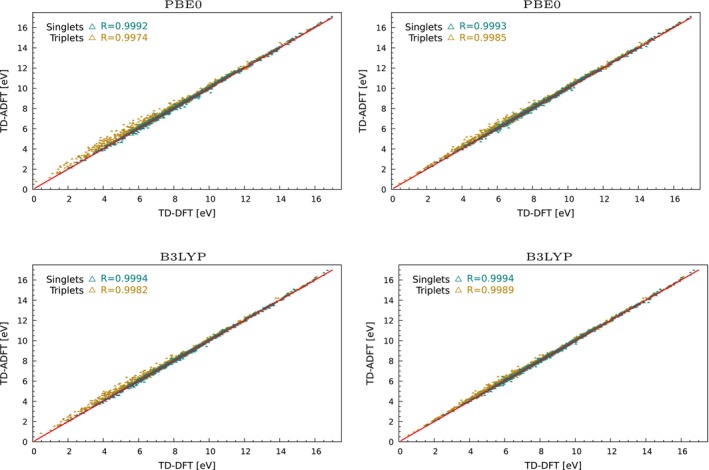
Linear correlation between TD‐DFT and HDA TD‐ADFT excitation energies for RPA (left) and TDA (right) global hybrid calculations.

For a more quantitative assessment of the HDA for global hybrid functionals we have calculated, analogous to the GGA validation, the first 30 singlet and triplet excitations with the HDA TD‐ADFT implementations of the PBE0 and B3LYP global hybrid functionals using the subsets Q1, Q3 and Q5 from the QUEST database. The corresponding correlation plots between TD‐DFT and HDA TD‐ADFT excitation energies are depicted in Figure 4. As these figures show good correlation between singlet TD‐DFT and TD‐ADFT excitations are obtained for both global hybrid functionals. Figure 6 shows that the corresponding MAEs and RMSE are in the same range as for the BLYP GGA functional. However, the MSEs are much smaller due to the non systematic deviations. This is a fundamental difference to the LDA and GGA TD‐ADFT implementation and is caused by the HDA. Whereas the agreement for HDA global hybrid singlet excitations is rather satisfying, much larger errors between TD‐DFT and TD‐ADFT are found for triplet excitations. This is immediately seen from Figure 4 by the larger spread for the triplet excitations represented by the dark yellow triangles. Figure 6 shows for these excitations MAEs well above 0.1 eV.

### 3.3 Range‐Separated Hybrid Functionals

**FIGURE 5:**
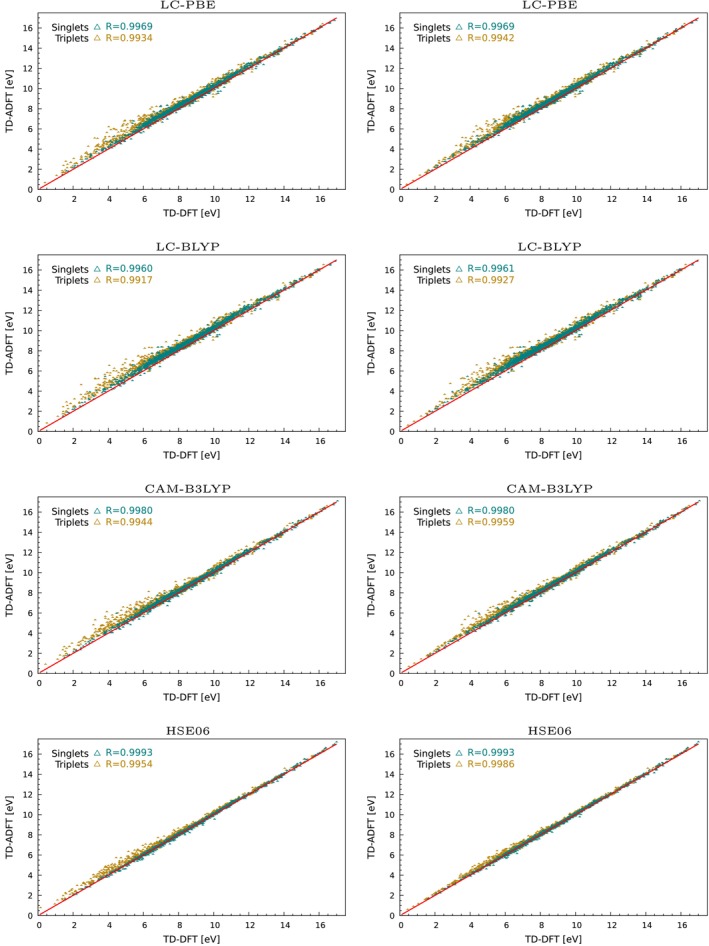
Linear correlation between TD‐DFT and TD‐ADFT excitation energies for RPA (left) and TDA (right) range‐separated hybrid calculations.

In order to extend the application range of TD‐ADFT, the HDA was adapted to range‐separated hybrid functionals, too. To this end, we have calculated the first 30 singlet and triplet excitations with the HDA TD‐ADFT implementations of the LC‐PBE, LC‐BLYP, CAM‐B3LYP and HSE06 range‐separated hybrid functionals considering the subsets Q1, Q3 and Q5 from QUEST database. In Figure 5 the correlation between TD‐DFT and HDA TD‐ADFT excitation energies are shown for the mentioned range‐separated hybrid functionals. A similar behavior as for the PBE0 and B3LYP global hybrid functionals is observed for LC‐PBE, LC‐BLYP and CAM‐B3LYP. In particular, a significant increased spread for triplet excitation is observed for all range‐separated functionals. Moreover, the corresponding MAEs, MSEs and RMSEs are significantly larger as for the global hybrids functionals. These results renders LC‐PBE and LC‐BLYP HDA TD‐ADFT calculations basically useless because the deviations due to the HDA are in the range of the intrinsic accuracy of TD‐DFT itself. CAM‐B3LYP HDA singlet excitation are at the borderline. Surprisingly, the inverse range‐separated hybrid functional HSE06 behaves similar to PBE0.

**FIGURE 6:**
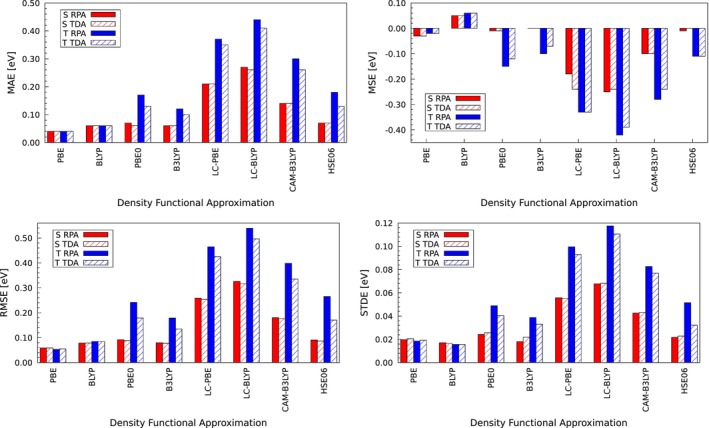
Mean average error (MAE), mean signed error (MSE), root mean square error (RMSE) and standard deviation (STDE) for PBE, BLYP, PBE0, B3LYP, LC‐PBE, LC‐BLYP, CAM‐B3LYP and HSE06 excitation energies.

To conclude the validation section we summarize in Figure 6 the MAEs, MSEs, RMSEs and STDEs of all investigated functionals. This figure underlines the excellent agreement of TD‐ADFT GGA excitations with their TD‐DFT counterparts. Similar accordance is found for the singlet excitations of global hybrid functionals (PBE0, B3LYP in Figure 6) employing HDA TD‐ADFT. On the other hand, HDA TD‐ADFT range‐separated results present larger deviations from the TD‐DFT excitation energies, particularly those calculated with LC‐PBE and LC‐BLYP functionals. We speculate that this arises from the fact that these functionals incorporate a larger portion of Fock exchange in comparison to global hybrids like PBE0 or B3LYP. Characteristic to all HDA results are the significantly larger deviations from TD‐DFT for the triplet excitations. Here further studies are needed.

Graphical error distributions of the validated functionals are given in Figure S2 in the Supporting Information. PBE and BLYP errors are for most of the molecules from the Q1, Q3 and Q5 sets from QUEST database between −0.1 and 0.1 eV and between −0.1 eV and 0.2 eV, respectively. For PBE0, B3LYP, CAM‐B3LYP and HSE06 this error range increases from −0.2 to 0.2 eV. Finally, the largest error ranges are found for the LC‐PBE and LC‐BLYP functionals with a span from −0.6 to 0.0 eV. Clearly, this renders the HDA unsuitable for these functionals. As a general trend, we observed that TD‐ADFT excitation energies are larger than the corresponding TD‐DFT ones.

### 3.4 Charge‐Transfer Excitations

We now turn to charge‐transfer excitations which are one of the main motivations behind our HDA implementation in TD‐ADFT. To this end, the sets Q6 and Q7 from the QUEST database with 30 well characterize CT excitations from 19 organic molecules are taken into account. The results of the TD‐ADFT calculations are shown in Table 1 together with the corresponding MAEs and MSEs with respect to CC3 reference values from the literature [70]. For comparison the MAEs and MSEs for TD‐DFT calculations are listed, too in Table 1. Because the CC3 reference calculations as well as the TD‐DFT calculations were performed with cc‐pVTZ basis set, we used the same basis set for our TD‐ADFT calculations in conjunction with the automatically generated GEN‐A2* auxiliary basis set. The catastrophic failures of GGA functionals for this type of excitations are immediately seen from the large MAEs for PBE and BLYP in Table 1. The corresponding MSEs reflect the severe underestimation of charge‐transfer excitation energies by GGA functionals. This is confirmed by comparing the individual PBE and BLYP excitations with the CC3 references. Note also the identical GGA MAEs and MSEs for TD‐ADFT and TD‐DFT. A plausible explanation for this failure of GGAs for charge‐transfer excitation was given by Maitra [79]. Because these excitations are mainly determined by transitions between unoccupied orbitals that have little to no spatial overlap with the occupied Kohn–Sham orbitals, the matrix elements of the pure exchange‐correlation kernels vanish. Therefore, the TD‐DFT and TD‐ADFT excitation energies reduce to the Kohn–Sham orbital energy difference which are usually much to small for GGAs.

**TABLE 1:** HDA TD‐ADFT charge‐transfer excitation comparison with CC3 reference results from the literature. For the TD‐ADFT calculations the cc‐pVTZ basis set in conjuntion with te GEN‐A2* auxiliary basis set was used.

		PBE	BLYP	PBE0	B3LYP	LC‐	LC‐	CAM‐	CC3^a^
						PBE	BLYP	B3LYP	
Aminobenzonitrile	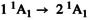	4.77	4.74	4.95	4.89	5.22	5.24	5.12	5.25
Aniline	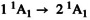	5.42	5.38	5.55	5.49	5.72	5.73	5.64	5.86
Azulene	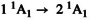	3.49	3.46	3.52	3.49	3.71	3.71	3.60	3.88
Azulene	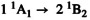	4.47	4.44	4.66	4.59	4.88	4.90	4.79	4.52
Benzonitrile	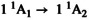	5.76	5.76	6.44	6.30	7.10	7.29	7.02	7.08
Benzothiadiazole	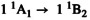	3.59	3.54	3.71	3.65	4.16	4.17	3.93	4.30
β−Dipeptide	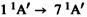	6.02	5.93	7.07	6.60	8.79	8.69	8.56	8.46
β−Dipeptide	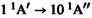	7.06	6.76	8.32	7.78	9.30	9.37	8.78	8.78
Dimethylaminobenzonitrile	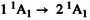	4.39	4.38	4.66	4.60	4.98	5.03	4.89	4.93
Dimethylaniline	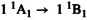	4.08	4.09	4.47	4.40	4.71	4.79	4.70	4.48
Dimethylaniline	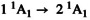	5.03	5.02	5.26	5.20	5.49	5.53	5.43	5.53
Dipeptide	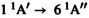	6.44	6.16	7.46	7.05	8.34	8.15	7.89	8.04
Hydrogen chloride	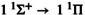	7.63	7.43	7.94	7.69	8.01	7.84	7.88	8.11
Nitroaniline	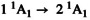	3.63	3.59	4.05	3.93	4.62	4.68	4.41	4.51
Nitrobenzene	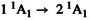	4.48	4.42	4.85	4.72	5.33	5.36	5.13	5.52
Nitrodimethylaniline	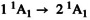	3.33	3.30	3.80	3.67	4.46	4.54	4.23	4.22
Nitropyridine N‐oxide	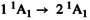	3.57	3.52	3.69	3.61	4.16	4.17	3.93	4.13
N‐Phenylpyrrole	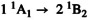	4.36	4.33	4.83	4.72	5.50	5.60	5.23	5.50
N‐Phenylpyrrole	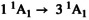	4.64	4.61	5.09	4.93	6.35	6.46	5.92	5.97
Phthalazine	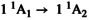	2.64	2.69	3.72	3.55	4.28	4.55	4.40	3.89
Phthalazine	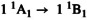	3.23	3.28	4.08	3.95	4.57	4.80	4.65	4.32
Quinoxaline	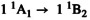	3.78	3.75	4.06	3.97	4.54	4.59	4.35	4.69
Quinoxaline	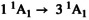	5.52	5.48	5.87	5.76	6.17	6.21	6.08	5.76
Quinoxaline	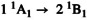	4.85	4.90	5.94	5.78	6.47	6.74	6.60	6.26
Twisted DMABN	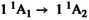	2.35	2.42	3.43	3.28	4.26	4.53	4.24	4.15
Twisted DMABN	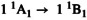	4.78	4.60	5.10	5.02	5.34	5.37	5.26	4.81
Twisted PP	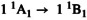	3.60	3.59	4.58	4.38	5.53	5.55	5.49	5.67
Twisted PP	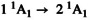	3.64	3.63	4.67	4.45	6.08	6.21	5.62	5.76
Twisted PP	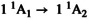	4.22	4.20	5.26	5.04	6.50	6.69	6.16	6.01
Twisted PP	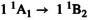	4.25	4.23	5.36	5.12	6.49	6.52	6.26	6.24
**TD‐ADFT**	**MAE (eV)**	**1.05**	**1.10**	**0.51**	**0.65**	**0.22**	**0.29**	**0.20**	
	**MSE (eV)**	**−1.02**	**−1.07**	**−0.46**	**−0.62**	**0.14**	**0.21**	**−0.02**	
**TD‐DFT**	**MAE (eV)**	**1.05**	**1.10**	**0.47**	**0.59**	**0.15**	**0.19**	**0.16**	
	**MSE (eV)**	**−1.02**	**−1.07**	**−0.40**	**−0.54**	**0.05**	**0.09**	**−0.06**	

*Note:* All values are in eV. Bold values show mean errors and are quality indicators for the used methodology (functional).

^a^ Reference [70].

To overcome this drawback the incorporation of Fock exchange into the density functional approximation is advocated. As Table 1 shows already with global hybrid functionals a significant reduction of the MAEs is observed. However, the errors with respect to the CC3 reference excitations are still well above 0.3 eV which is not acceptable. Please note that the difference between HDA TD‐ADFT and TD‐DFT MAEs are in the range of 0.05 eV. This is an order of magnitude smaller than the MAEs for PBE0 and B3LYP. Thus, HDA seems promising for improving charge‐transfer excitation energies. Additional improvements are obtained if range‐separated hybrid functionals such as LC‐PBE, LC‐BLYP and CAM‐B3LYP are used. At this point it is important to remember that LC‐PBE and LC‐BLYP show unacceptably large MAEs between HDA TD‐ADFT and TD‐DFT. Therefore, we recommend CAM‐B3LYP for TD‐ADFT studies that include charge‐transfer excitations. We also note that HDA in its current form is only reliable for singlet excitations.

### 3.5 HDA Benchmarking

**FIGURE 7:**
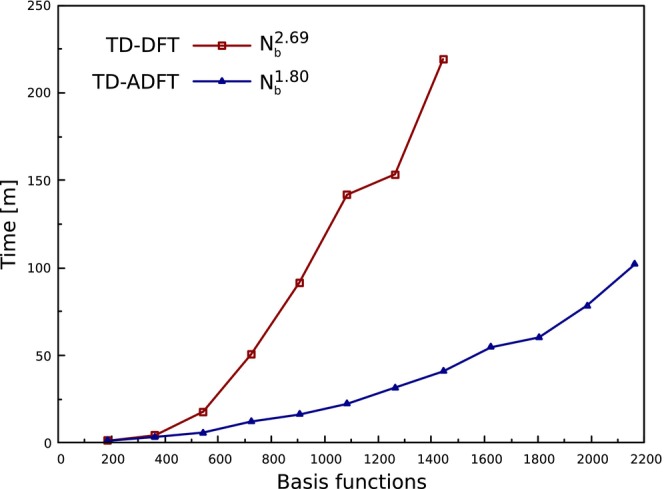
Comparison of serial computational timings for the lowest 10 excitations energies of linear alkane chains between HDA TD‐ADFT (deMon2k) and TD‐DFT (Orca) employing PBE0 and the 6‐31G* basis set. For the density fitting in deMon2k the automatically generated GEN‐A2* auxiliary basis set was used.

In order to assess the performance of our HDA TD‐ADFT implementation, linear alkane chains were used to benchmark serial timings. The reported HDA TD‐ADFT timings include the $\Delta_{ai}^{GH}$ calculation and the Davidson diagonalization for the first 10 singlet excitation energies. We used the PBE0/6‐31G*/GEN‐A2* level of theory for these benchmark calculations. The benchmark calculations were carried out with an Intel(R) Core(TM) i5‐3570 CPU@3.40 GHz with 3 GB of RAM. Figure 7 compares the HDA TD‐ADFT timings with corresponding TD‐DFT timings from Orca. Most notable is the sub‐quadratic scaling of HDA TD‐ADFT. This is in accordance with previous benchmark results by Hernández‐Segura et al. [28] for GGA functionals despite the now included calculations of $\Delta_{ai}^{GH}$. We attribute this to the discussed $\Delta_{ai}^{GH}$ integral calculation and the preserved alignment between ERIs and excitation vectors in the Davidson diagonalization. Another advantage of HDA TD‐ADFT is its low RAM consumption. In Figure 7 TD‐DFT calculations were only possible up to ${\text{C}}_{80}{\text{H}}_{162}$ whereas HDA TD‐ADFT calculations were feasible up to ${\text{C}}_{120}{\text{H}}_{242}$ with the available 3 GB of RAM.

## 4 Conclusions

The working equations for HDA TD‐ADFT are derived and an efficient algorithm for the calculation of the HDA specific matrix elements is presented. The validation of global hybrid HDA TD‐ADFT singlet excitation energy calculations considering the test set Q1, Q3, and Q5 of the QUEST database show good agreement with respect to the corresponding TD‐DFT calculations. Corresponding results for range‐separated hybrid functionals are less convincing. Only for CAM‐B3LYP a fair agreement between HDA TD‐ADFT and TD‐DFT was found. However, all HDA triplet excitations show unacceptable large deviation from corresponding TD‐DFT results. We suspect a systematic failure of HDA for triplet excitations. This is currently under further investigation in our laboratory.

Our calculations of singlet charge‐transfer excitations show that the catastrophic errors of GGA functionals can be dramatically reduced by hybrid functionals within the HDA. Particularly satisfying is the performance of CAM‐B3LYP in the framework of HDA TD‐ADFT. In combination with the outstanding computational performance and low‐order scaling of HDA TD‐ADFT calculations this approach is most promising for large scale excitation energy calculations. However, in its current form it is limited to singlet excitation only.

## Conflicts of Interest

The authors declare no conflicts of interest.

## Supporting information




**Data S1:** Supporting Information.

## Data Availability

The data that support the findings of this study are available on request from the corresponding author. The data are not publicly available due to privacy or ethical restrictions.
