# Supplementary material for: Hybrid Diagonal Approximation in Time‐Dependent Auxiliary Density Functional Theory
Source: J Comput Chem. 2025 Sep 2;46(23):e70210. doi: 10.1002/jcc.70210 (PMC12404025; doi:10.1002/jcc.70210)
Supplement: Supplementary file 1 — Data S1: Supporting Information. [file JCC-46-0-s001.pdf]

Supporting Information for:
Hybrid Diagonal Approximation in Time-Dependent Auxiliary Density Functional Theory

Kevin O. Pérez-Becerra,*,† Jesús Naín Pedroza-Montero,‡,¶ Mark R. Pederson,¶ Luis I. Hernández-Segura,† and Andreas M. Köster*,†

†*Departamento de Química, Cinvestav, Avenida Instituto Politécnico Nacional 2508, CDMX C.P. 07360, México*

‡*Physics Department, Central Michigan University, Mt. Pleasant, Michigan 48859, USA*

¶*Department of Physics, University of Texas at El Paso, El Paso, Texas 79968, USA*

E-mail: kevino.perez@cinvestav.mx; akoster@cinvestav.mx

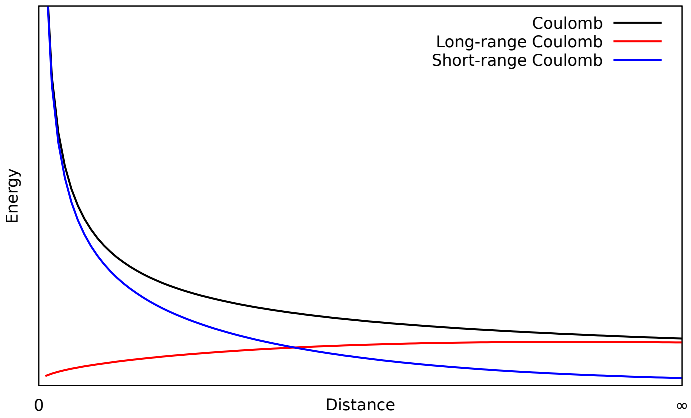

Figure S1: Coulomb operator partition with separation ratio parameter $\omega = 0.40$.

Exchange-correlation energy expressions for the RS-B88, RS-PBE and HSE functional families in ADFT:[1]

$$E_{xc}^{RS-B88}[\tilde{\rho}] = -c_F\left(\alpha\sum_{i,j}^{occ}\sum_{\bar{k},\bar{l}}\langle\psi_i\psi_j||\bar{k}\rangle G_{\bar{k}\bar{l}}^{-1}\langle\bar{l}||\psi_i\psi_j\rangle + \beta\sum_{i,j}^{occ}\sum_{\bar{k},\bar{l}}\langle\psi_i\psi_j\underline{||}\bar{k}\rangle \underline{G}_{\bar{k}\bar{l}}^{-1}\langle\bar{l}\underline{||}\psi_i\psi_j\rangle\right) + E_x^{SR-B88}[\tilde{\rho}] + \gamma\Delta E_x^{B88}[\tilde{\rho}] + \delta E_c^{LYP}[\tilde{\rho}] + (1-\delta)E_c^{VWN5}[\tilde{\rho}] \quad (1)$$

$$E_{xc}^{RS-PBE} = -c_F\left(\alpha\sum_{i,j}^{occ}\sum_{\bar{k},\bar{l}}\langle\psi_i\psi_j||\bar{k}\rangle G_{\bar{k}\bar{l}}^{-1}\langle\bar{l}||\psi_i\psi_j\rangle + \beta\sum_{i,j}^{occ}\sum_{\bar{k},\bar{l}}\langle\psi_i\psi_j\underline{||}\bar{k}\rangle \underline{G}_{\bar{k}\bar{l}}^{-1}\langle\bar{l}\underline{||}\psi_i\psi_j\rangle\right) + E_x^{SR-PBE}[\tilde{\rho}] + E_c^{PBE}[\tilde{\rho}] \quad (2)$$

$$E_{xc}^{RS-HSE} = -c_F\left(\sum_{i,j}^{occ}\sum_{\bar{k},\bar{l}}\langle\psi_i\psi_j||\bar{k}\rangle G_{\bar{k}\bar{l}}^{-1}\langle\bar{l}||\psi_i\psi_j\rangle - \sum_{i,j}^{occ}\sum_{\bar{k},\bar{l}}\langle\psi_i\psi_j\underline{||}\bar{k}\rangle \underline{G}_{\bar{k}\bar{l}}^{-1}\langle\bar{l}\underline{||}\psi_i\psi_j\rangle\right) + (1-c_F)E_x^{PBE}[\tilde{\rho}] + c_F E_x^{LR-PBE}[\tilde{\rho}] + E_c^{PBE}[\tilde{\rho}] \quad (3)$$

For this work γ and δ parameters in the RS-B88 functional template are set to 0 and 0.81 respectively. Using other α, β, γ, δ and ω parameters in the RS-B88 functional template yields range-separated functionals not considered in this work like rCAM-B3LYP,[2] CAM-QTP00[3] and CAM-QTP01.[4]

Table S1: Functional parameters.

	c_F	α	β	ω
**LC-PBE**	1.00	0.00	1.00	0.30
**LC-BLYP**	1.00	0.00	1.00	0.33
**CAM-B3LYP**	1.00	0.19	0.46	0.33
**HSE06**	0.25	0.00	1.00	0.11

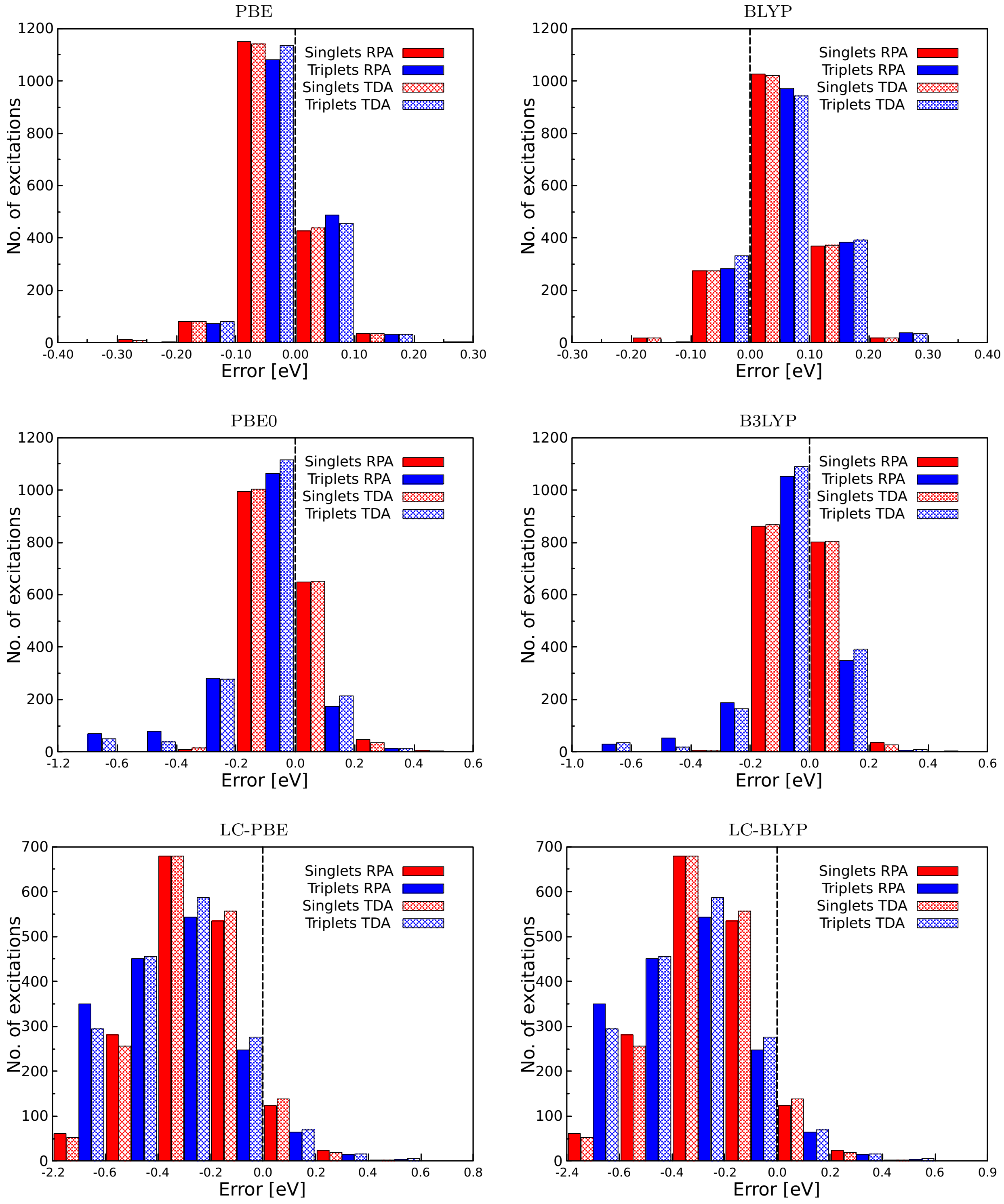
PBE
BLYP
PBE0
B3LYP
LC-PBE
LC-BLYP
Singlets RPA
Triplets RPA
Singlets TDA
Triplets TDA
No. of excitations
Error [eV]

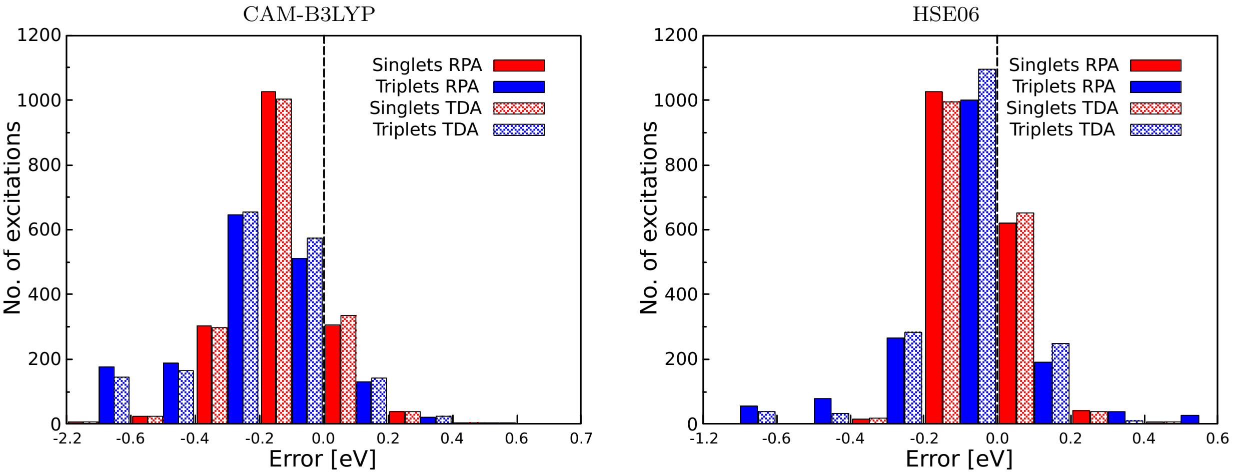

Figure S2: Histograms for all excitation energy calculations from subset Q1, Q3 and Q5.

References

(1) Delesma, F. A. *Range-Separated Hybrid Functionals in Auxiliary Density Fucntional Theory*; PhD Thesis Cinvestav, 2020; p 43.

(2) Cohen, A. J.; Mori-Sánchez, P.; Yang, W. Development of exchange-correlation functionals with minimal many-electron self-interaction error. *J. Chem. Phys.* **2007**, *126*, 191109.

(3) Verma, P.; Bartlett, R. J. Increasing the applicability of density functional theory. IV. Consequences of ionization-potential improved exchange-correlation potentials. *J. Chem. Phys.* **2014**, *140*, 18A534.

(4) Jin, Y.; Bartlett, R. J. The QTP family of consistent functionals and potentials in Kohn-Sham density functional theory. *J. Chem. Phys.* **2016**, *145*, 034107.
